# Measuring the Chemical and Cytotoxic Variability of Commercially Available Kava (*Piper methysticum* G. Forster)

**DOI:** 10.1371/journal.pone.0111572

**Published:** 2014-11-03

**Authors:** Amanda C. Martin, Ed Johnston, Chengguo Xing, Adrian D. Hegeman

**Affiliations:** 1 The Department Horticultural Science, Plant Biological Sciences Graduate Program, Department of Plant Biology, and Microbial and Plant Genomics Institute, University of Minnesota, St Paul, MN, United States of America; 2 Alia Point ‘Awa Nursery, Pepe’ekeo, Hawai’i, United States of America; 3 Department of Medicinal Chemistry, University of Minnesota, Minneapolis, MN, United States of America; University of Washington, United States of America

## Abstract

Formerly used world-wide as a popular botanical medicine to reduce anxiety, reports of hepatotoxicity linked to consuming kava extracts in the late 1990s have resulted in global restrictions on kava use and have hindered kava-related research. Despite its presence on the United States Food and Drug Administration consumer advisory list for the past decade, export data from kava producing countries implies that US kava imports, which are not publicly reported, are both increasing and of a fairly high volume. We have measured the variability in extract chemical composition and cytotoxicity towards human lung adenocarcinoma A549 cancer cells of 25 commercially available kava products. Results reveal a high level of variation in chemical content and cytotoxicity of currently available kava products. As public interest and use of kava products continues to increase in the United States, efforts to characterize products and expedite research of this potentially useful botanical medicine are necessary.

## Introduction

Kava (*Piper methysticum* G. Forster) is the name of a plant and drink that is prepared traditionally by macerating its roots in cool water or coconut water [1]. It has been used for many centuries in the South Pacific and Hawaii for social ceremonies, relaxation, medicine, and a multitude of other purposes [1]. More recently, standardized kava extracts, containing 30% active constituents, have been used globally as an anxiolytic [2], [3]. Additionally, a tight inverse correlation between high rates of kava consumption and low incidences of cancer for populations in the South Pacific has been reported [4]. Subsequent studies have shown that kava displays cancer preventive properties [5]–[8].

There are about 200 different cultivated varieties of kava [9], each with a unique chemotype that produces specific physiological and psychoactive effects [10]–[13]. The active constituents are chemically classified as kavalactones and six (kawain, dihydrokawain, methysticin, dihydromethysticin, yangonin, and desmethoxyyangonin) constitute the primary chemicals that are responsible for individual cultivars’ unique chemotypes [1], [14]–[16].

A 2002 the Kava Act passed in Vanuatu established four classes of kava cultivars: noble, which have a long history of safe use as traditional drink; medicinal, which have long been used by traditional herbalists in the South Pacific and are banned as export commodities; ‘Tu dei’, which have a very strong effect that lasts two days; and ‘Wichmanni’ or wild varieties [9], [17], [18]. Cultivars from the noble class are typically used to prepare kava extract as they have the optimal therapeutic chemotype. Cultivars belonging to other classes have been reported to have overpowering and unpredictable effects causing symptoms such as nausea and headaches [19], [20]. Kava’s active constituents are primarily located in its roots; other plant parts such as stems and leaves should not be used in extract preparations [1]. Traditional kava is prepared using a 100% aqueous solvent, which results in a drink containing an average of 0.3–20% kavalactone content [1]. Commercial manufacturers use up to 100% ethanol or acetone in the extraction process resulting in up to 70% kavalactone content in the final product [1], [20]. Studies have shown a difference between traditionally prepared extracts and those prepared with ethanol both in cytotoxicity and chemical composition [21]–[25]. In addition to those described above, other potential sources of variation in kava products include, contamination of raw kava materials, impurities, post-harvest handling and storage procedures (drying, whole vs. ground material, humidity, temperature), age of harvested kava plants, mixture and quality of cultivar(s) used [17], [26]–[28] Kava is distributed in variable forms, including dry powder, freeze-dried, liquid tincture, and capsule, making it difficult to know exactly which cultivar(s), plant part(s), extraction solvent(s), and other factors were used in the preparation [26].

Due to reports linking modern kava consumption to individual cases of hepatotoxicity, kava was banned in the European Union and Canada in 2003, voluntarily recalled in Australia in 2003, and included on the United States Food and Drug Administration (US FDA) consumer advisory list in March of 2002 [14], [29]–[32]. These bans and advisories have hindered research on kava as an alternative anti-anxiety and cancer preventive medicine [17]. Despite its presence on the US FDA consumer advisory list for the past decade, the extrapolation of export data from the kava producing nations Fiji, The Republic of Vanuatu, and Tonga to the US (Table 1) indicates that kava imports to the US, which are not publicly reported, are presumably both increasing and of a fairly high volume [33]–[36].

**Table 1:** Kava exports from Fiji, Tonga and Vanuatu: 2008 through 2013^a^.

	Total metric tons exported (subset exp. to US)		
Year	from Fiji^33^	from Tonga^34^	from Vanuatu^35^
2008	184 (93)	27^36^	356^36^
2009	212 (123)	38.9 (0.7)	485^36^
2010	244 (91)	61.6 (29.2)	498
2011	276 (95)^b^	68.6 (42)	734
2012	NA	117 (80)	643
2013	NA	NA	558^c^

a Kava exports are reported in metric tons where available from 2008 through 2013. The subset of exports to the United States is given parenthetically next to each total export figure where available. NA indicates that the data were not available for that year from the sources cited.

b Represents exports for January through November of 2011.

c Represents exports for January through August 2013.

**Figure 1:**
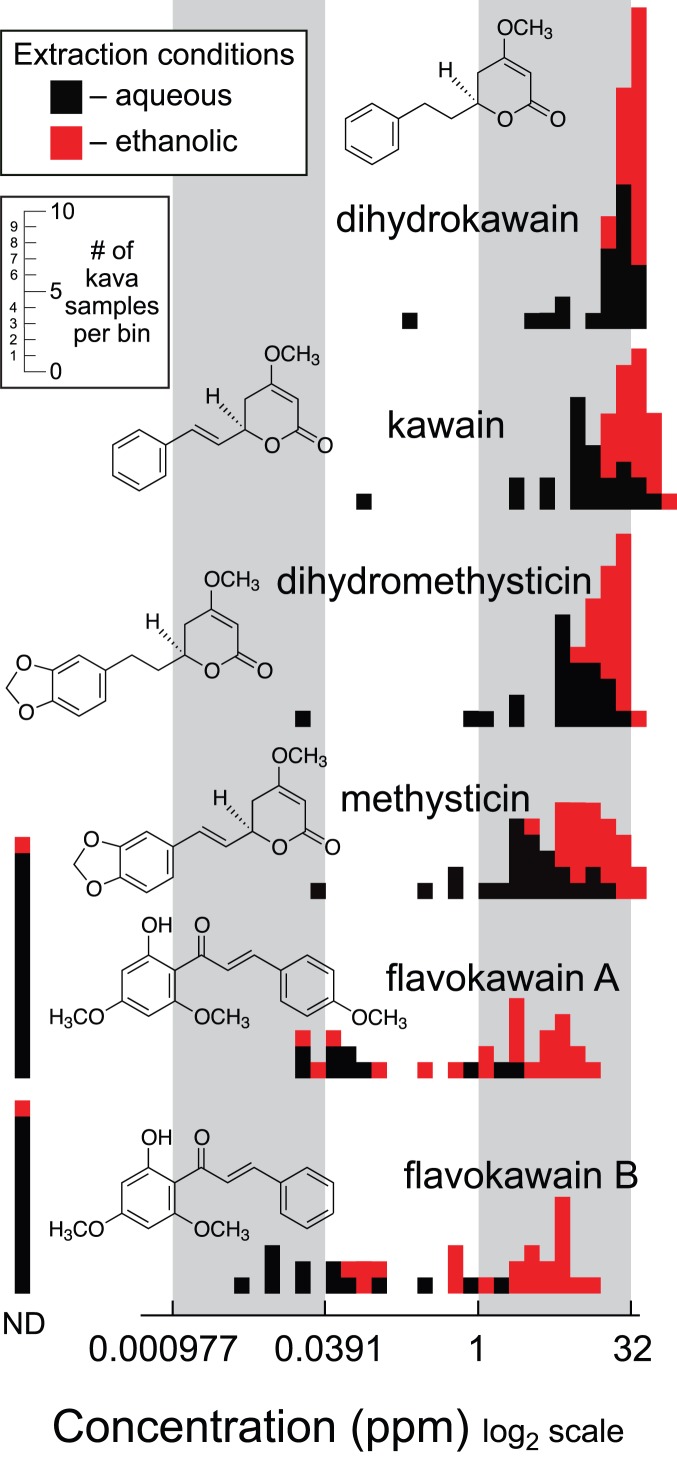
Histograms showing the distribution of concentrations of compounds found in commercial kava preparations. Kava samples were extracted with both 100% water (black) and 95% ethanol (red). The six compounds shown were quantified in each extract by LC-MS and the resulting concentrations in part per million are displayed histogramatically. Distributions were normalized by display on a log_2_ scale. The inset y-axis scale indicates the numbers of kava samples in each bin. Measurements designated as not detected (ND) were below the limits of detection (LOD (*s/n*<3) for each analysis, which were typically 0.0005 to 0.001 ppm depending on variation in signal to noise from sample to sample.

There are many hypothesized mechanisms potentially linking kava consumption to hepatotoxicity [3], [14], [25], [26], [32], [37]–[43]. We intended to measure the overall variation in cellular toxicity and chemical composition among the large volume of diverse kava products currently available. Only six kavalactones have been intensively studied [2], [16], [18], [44], [45] making it necessary to assess the complete pool of extracted compounds. We performed metabolic fingerprinting; a metabolomics technique that facilitates comparisons based on global metabolite patterns of whole extracts [46]. We used ultra-performance liquid chromatography-electrospray ionization-time-of-flight-mass spectrometry (UPLC-ESI-TOF-MS) to fingerprint replicate aqueous and 95% ethanolic extracts of 25 commercial kava products (Table S1). We also quantified six compounds found in kava that may be associated with either the medicinal or negative cytotoxic effects of modern kava usage: kawain (K); dihydrokawain (DHK); methysticin (M); dihydromethysticin (DHM); flavokawain A (FLK A); and flavokawain B (FLK B) [24], [37]–[39] (Figure 1). Absolute quantification was performed using pure standards and a UPLC-single quadrupole mass spectrometer (MS). Finally, we determined the cytotoxicity levels of each extract in cell viability assays towards human lung adenocarcinoma A549 cancer cell line.

## Results and Discussion

Metabolic fingerprinting experiments measured three aspects of chemical variation: reproducibility of replicate extractions of individual products; differences between using 100% water or 95% ethanol as the extraction solvent; and the overall variation among the set of kava products tested. Similarly to previous quantitative studies of compounds from kava, we observed a high level of reproducibility of replicate extractions of material from individual kava sources [45]. Principal component analysis showed that replicate extractions from the same kava source are tightly clustered (Figure S1). Moreover, there were small standard errors (average standard error 12.6%) from the absolute quantification measurements of K, DHK, M, DHM, FLKA, and FLKB (Tables S2 and S3). These results provide evidence that there is consistency in the material contained within a single batch of kava from any given source.

Extract chemical composition was strongly influenced by extraction solvent. Metabolic fingerprints from aqueous and ethanolic extracts plotted in principal component space formed two distinct groups driven by extraction solvent where the use of either 100% water or 95% ethanol was responsible for 71.1% of the variation among all samples explained by PC1 (Figure S2). The detected ion *m/z*, retention time pairs that contribute the most to the loadings for PC1 were 315.1132 *m/z*, 9.1823 min and 285.1021 *m/z*, 9.4699 min, which correspond to the masses and retention times of FLKA and FLKB, respectively. Compound quantification showed that extracts prepared with 95% ethanol resulted in higher yields and greater consistency among replicates, compared with extracts prepared with 100% water. This result is similar to previous studies that found water produced kava extracts with decreased compound concentrations compared to extracts prepared with ethanol [22], [23]. Specifically, K, DHK, M, and DHM concentrations were 1.5–5x higher in samples extracted with 95% ethanol than in those extracted with 100% water. The concentrations of FLKA & FLKB were up to fifty times higher in samples extracted by 95% ethanol than in those extracted with 100% water although a significant number of the water extracts contained concentrations of FLKA or FLKB that were below detectable limits (Figure 1). Extracts prepared with 95% ethanol consistently contained greater quantities of FLKA and FLKB than corresponding water extracts, and were highly variable across kava products (ranging from undetectable concentrations up to 14.7 ppm; Figure 1).

While extraction solvent was the most influential variable affecting the observed chemical composition, significant variation in the concentrations of K, DHK, M, and DHM for identically prepared extracts was observed from different source materials. This variation was even more dramatic in regard to the concentration of FLKA and FLKB. The variation in chemical composition was further reflected by the differences in cytotoxicity observed for each commercial kava product extract.

**Figure 2:**
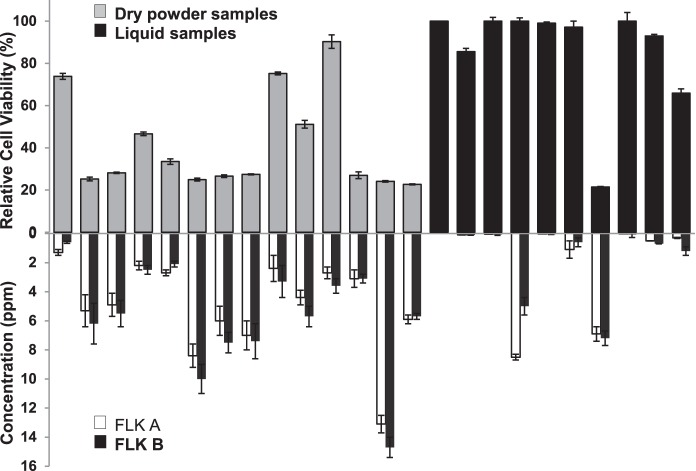
Comparison of relative cell viability to flavokawain (FLK) A and B concentrations. **Top.** Relative cell viability of human lung adenocarcinoma A549 cancer cell line after 48-hour incubation with ethanolic kava extracts at 75 µg/mL. Samples are organized according to kava preparation type with the gray bars representing the dry power samples organized from coarse grind on the left to very fine grind on the right with the last three dry powder samples (P, Z, and V) being instant freeze-dried kava. Black bars represent liquid samples. **Bottom.** Concentration of two potentially cytotoxic compounds found in kava (white bars: FLKA and black bars: FLKB respectively). Error bars represent standard error of 3–4 replicates.

Cytotoxicity assays against human lung adenocarcinoma A549 cancer cell line with aqueous extracts from all 25 commercial kavas showed no toxicity at any concentration measured up to 500 µg/mL. This result is similar to previous studies indicating that aqueous extracts have low to no cytotoxic effect [20]. In contrast, identically prepared ethanol extracts from different commercial sources varied greatly in their relative cytotoxicity at all concentrations measured 37.5, 75 (shown in Figure 2 top), and 150 µg/mL. Ethanol extracts prepared from commercial kava sources K, M, N, O, W, Y, DD, and EE exhibited very low cell toxicity at all concentrations, indicated by a relative cell viability level of greater than 90%. In contrast, ethanolic extracts from G, H, P, Q, R, S, V, Z, and BB, displayed the highest levels of toxicity, with a relative cell viability level of less than 30%. Cytotoxicity levels at these three discreet extract concentrations varied over a wide range similarly to the variation observed in extract chemical composition, especially in regard to FLKA and FLKB.

We observed a moderate correlation between the concentrations of FLKA & FLKB (log_2_ normalized) and the relative cytotoxicity across the sampled kava products. High concentrations of the flavokawains generally mirrored lower relative cell viability (Figure 2). Some samples, however, deviated from this correlation, specifically N and BB, which have medium concentrations of FLKA and FLKB and display low and high cytotoxcity, respectively. Scatterplots of these data revealed that concentrations of FLKA and FLKB correlated similarly to cytotoxicity level with R^2^ values equal to 0.68 and 0.69, respectively for extracts prepared at 75 µg/mL and 0.78 and 0.77, respectively for extracts prepared at 150 µg/mL (Figures S3 and S4). Based on these correlation values, clearly, FLKA and FLKB are likely the major but not the solely compounds responsible for the extract toxicity. However, additional reports of flavokawain toxicity, including measured IC_50_ levels for FLKA and FLKB of 13±1.1 and 6.6±0.1 ppm, respectively against Hepa 1c1c7 liver cells [24], and 57% growth inhibition of bladder T24 tumor cells by FLKA [8] warrant further exploration of the link between flavokawains and kava’s cytotoxicity [21], [22].

We also generated a non-polar flavokawain enriched kava fraction to determine the IC_50_ values of 48-hour toxicities against hepatocytes from mouse, rat, and monkey. We measured IC_50_ values for this FLK rich fraction of 57±9, 45±4 and 49±6 µg/mL, for mouse, rat, and monkey hepatocytes, respectively. In contrast polar and medium polarity fractions and whole traditionally prepared kava had non-detectable IC_50_ values greater than 400 µg/mL in all three cell types.

For each compound, K, DHK, M, and DHM there was no obvious association between concentration and relative cell viability, although extracts with higher overall concentrations of all six compounds resulted in lower relative cell viability. This trend suggests that K, DHK, M, and DHM are less likely to be involved in the specific mechanism(s) of cytotoxicity.

Additional reports of cytotoxic compounds found in kava describe one additional flavokawain, distinct from FLKA and FLKB with the following chemical formula and exact mass C_17_H_16_O_5,_ 300.0998 (FLK C) [35] and three alkaloids found in kava leaves with the following chemical formulae and exact masses: C_14_H_17_NO_2_, 231.1259 (awaine); C_16_H_17_NO4, 287.1157 (pipermethystine); C_16_H_17_NO_5_, 303.1106 (3α, 4α-epoxy-5β-pipermethystine) [47]. These alkaloids may have been present in commercial kava products produced by European companies leading up to the European ban of kava in 2003 [18] We looked for patterns between cytotoxicity level and the presence of these potentially toxic compounds using M^+^H extracted ion chromatograms. Overall, only a peak corresponding to FLKC was detectable above the limit of detection (*s/n*>3), where the relative intensity of this peak was higher in extracts with higher toxicities. Additional experiments are necessary to understand how these compounds interact with FLKA and FLKB to produce extract cytotoxicity [17], [18], [26], [28]. Regardless of the precise cause of cytotoxicity it is clear that tremendous variation exists in the chemical composition and resulting toxicity of commercially available kava products.

## Conclusions

Kava export data show that in spite of bans and warnings, consumption of unregulated kava products appears to be increasing. Our analysis shows that the assortment of commercially available kava products vary widely in chemical composition and cytotoxicity level. Certain kava cultivars and preparation methods may produce products that vary broadly in both their toxicity and their efficacy and thus a rapid and easily applied method to characterize and classify kava products would be beneficial to the consumer. Disregarding kava and its potential use as an anxiolytic or for cancer preventive ignores the great potential societal benefits of the rational and informed medicinal use of this plant.

## Materials and Methods

### Solvents and reagents

HPLC grade solvents from Sigma Aldrich (St. Louis, MO, USA) were used including: acetonitrile, dimethyl sulfoxide (DMSO), formic acid, ethyl acetate, 95% ethanol, and hexanes. Reverse osmosis deionized glass distilled water was obtained in house using a Thermo Scientific Barnstead B-pure filter and Distinction water still model D4000 (Bibby Scientific Limited, Stone, Staffordshire ST15 0SA, UK). Standard kava compounds were purified from Gaia Herb (Brevard, NC, USA) commercial Kava extract. Commercial kava samples were obtained from a variety of sources (Table S1).

### Kava extraction

Kava samples from 25 different sources were classified as either powder (P) or liquid (L) (Table S1). Four extraction methods were used; methods I and II for powder samples and methods III and IV for liquid samples. The method details are as follows: Method I: 10 mL of room temperature water was added to 5 grams of powdered kava, shaken for 2 hrs, centrifuged to remove insoluble material and the supernatant evaporated to dryness and re-dissolved in water at a concentration of 1.5 mg of residue per mL. Method II: the same as I, except 95% ethanol was used in place of water. Method III: 200 µL of liquid kava sample was dried *in vacuo*, reconstituted in 500 µL of water and then adjusted to a concentration of 1.5 mg of residue per mL with additional water. Method IV: samples were directly diluted to 1.5 mg/mL with 95% ethanol. For each extraction method four replicates per sample were prepared for analysis. Extract yield was determined gravimetrically by evaporating 500 µL of extract to dryness using a Savant model SVC-200 H SpeedVac concentrator (Farmingdale, NY, USA). Extracts were normalized to 1.5 mg/mL for experiments and stored at 4°C in the dark for no more than a week prior to LC/MS analysis. All extractions were carried out at room temperature (approximately 25°C).

### Metabolic fingerprinting

Metabolic fingerprints were generated using C_18_-reversed-phase ultra-performance liquid chromatography-positive electrospray ionization-time-of-flight mass spectrometry (UPLC-ESI(+)-TOF-MS) carried out on a UPLC-TOF LCT Premier XE mass spectrometer fitted with an autosampler with a sample vial block maintained at 4°C (Acquity, Waters, Milford MA, USA). The following MS conditions were used: full scan mass scan range: 100–1000 *m/z*, W analyzer mode, extended dynamic range, 0.1 s scan time, desolvation temperature 350°C, desolvation nitrogen flow rate: 7.0 L/min, capillary voltage: 2900 V, sample cone voltage: 30 V, source temperature: 120°C. Separations were carried out on a reversed-phase C_18_ HSS T_3_ 1.8 µm particle size, 2.1×100 mm column (Waters). Column temperature was 50°C, solvent flow rate 0.3 mL/min, injection volume 5 µL. A 14-minute gradient using mobile phases A: 0.1% formic acid in water and B: 0.1% formic acid in acetonitrile was run according to the following gradient elution profile: initial, 10%; 3 minutes, 50% B; 8 minutes, 60% B; 13 minutes, 98% B; 14 minutes, 98% B. A 7-minute wash cycle was run between every sample and monitored for the absence of carryover. MassLynx version 4.1 (Waters) was used for data collection and visualization. Sample analysis order was randomized across the entire sample set.

### Feature detection and multivariate statistical analysis

LC-MS files were processed using MarkerLynx version 4.1 software (Waters) for feature detection using the following parameters: mass tolerance: 0.01 Da; peak width at 5% height: 0.2 s; intensity threshold: 2000 counts; mass window: 0.05 Da; retention time window: 0.20 s. Following feature detection the feature lists were imported into *Analyst* version 7.5 software (Genedata, Lexington, MA, USA). Feature lists were inspected and a feature was considered to be real if it was present in greater than 75% of replicate samples with similar intensity in all replicates**.** Once highly confident feature lists were obtained, principle component analysis (PCA) was performed.

### Absolute quantification

Absolute quantification was performed using a UPLC-single quadrupole mass spectrometer (Waters). Independent standard curves were generated for six compounds (K, DHK, M, DHM, FLKA, and FLKB) found in kava. Mixtures of pure standards were made in seven concentrations from 0.05 ppm to 100 ppm; four technical replicates were completed to account for chromatographic drift and ionization variability. After LC method optimization, standard curves were generated in selected ion recording (SIR) mode with the following retention time windows: 0–3.5 min: scan 220–700 *m/z*; 3.5–6.5 min: 275±2 *m/z*; 3.5–6.5 min: 277±2 *m/z*; 4.0–7.0 min: 231±2; 4.5–7.5 min: 233±2 *m/z*; 7.5–8.0 min scan 220–700 *m/z*; 8–11 min: 315±2 *m/z*; 9–12: 285±2 *m/z*; 12.0–14.0 min: scan 220–700 *m/z*. A cone voltage of 40 V was used to disfavor non-covalent compound dimerization in ESI^+^ mode. The liquid chromatography and column parameters are identical to those used for the metabolic fingerprinting. Standard curves were linear up to 50 ppm. Samples were analyzed using the same LC-MS method with three or four replicates in most cases, although for six samples (N, IV; X, III; Y, IV; BB, III&IV; CC, II) and four samples (J, IV; M, IV; O, IV; X, IV) only two or one replicates were suitable for the final quantification, respectively. The limit of detection was set at concentrations corresponding to a signal-to-noise ratio of 3 to 1; peaks occurring below this threshold are not detected (ND). The limit of quantification was set at a signal-to-noise ratio of 10 to 1. The MassLynx™ application manager QuanLynx™ (Waters) was used to assist with automatic integration and of this large dataset. All integrations were visually inspected and manually adjusted to ensure consistent and accurate quantification.

### Kava fractionation and characterization

Fractionation of commercial kava purchased from Gaia Herb (Brevard, NC, USA) was performed as described previously [7] with normal phase silica gel chromatography generating three modalities – fraction A (hydrophilic), B (medium polarity), and C (lipophilic). Briefly, 300 mL, net weight, of kava residue was mixed with silica gel (300 g). Ethanol and water were removed by vacuum. This silica gel with adsorbed kava residue was subjected to coarse chromatographic separation using a 750-gram pre-packed silica gel cartridge. The elution method was 28% ethyl acetate and 72% hexane 5 column volumes, followed by 90% ethyl acetate and 10% hexane, 4.1 column volumes, and then 35% methanol and 65% ethyl acetate, 5.5 column volumes. Different eluents were analyzed by TLC and the desired eluents were combined with solvent removed to generate fractions A, B, and C. The fractionation process was automated and monitored by the Biotag Separation System. Each individual fraction was analyzed by ^1 ^H^–^NMR and HPLC to confirm the success of fractionation.

### Cytotoxicity test

Cytotoxicity tests were performed on fractions A, B, & C and different kava extracts. Extracts were dried and reconstituted in DMSO at a concentration of 10 mg/mL. From these stock solutions working solutions of 150, 75, and 37.5 µg/mL were prepared from each extract. Their cytotoxicity against human lung adenocarcinoma A549 cancer cells (American Type Culture Collection CCL-185) were evaluated by following our established procedures [48]. Briefly, A549 cells were plated in a 96-well plate (2.5×103 cells/well). The cells were treated with kava extracts with 0.5% DMSO in the final cell media (cells treated with media containing 0.5% DMSO served as a control). After 48 h of treatment, the relative cell viability in each well was determined by using CellTiter-Blue cell viability assay kit (Promega, CA). Two biological repeats with three replicates per experiment were performed.

## Supporting Information

Figure S1 **Principal components analysis (PCA) of commercial kava preparations.** Dry ground kava was extracted with water. Replicate extractions of the same material form clusters identified by drawn circles. The percent of variation explained by each principal component is shown along the appropriate axis.
(EPS)

Figure S2 **Principal components analysis (PCA) of commercial kava preparations.** Dry ground kava was extracted with water (blue) and ethanol (red). Replicate extractions of the same material form tight clusters. Secondary groups identified by drawn circles are formed based on the extraction solvent used; where the large amount of variation explained by Eigenrow 1 (PC1) is due to the use of either water or ethanol. The percent of variation explained by each principal component is shown along the appropriate axis.
(EPS)

Figure S3 **Correlation between relative cell viability and Flavokawain A concentration.** Relative cell viability of human cancer cells after 48-hour incubation with kava extracts at 75 µg/mL (red, circles) and 150 µg/mL (blue squares) is plotted to sample FLK A concentrations (log_2_ normalized) with R^2^ values shown at the top for each extract concentration.
(EPS)

Figure S4 **Correlation between relative cell viability and Flavokawain B concentration.** Relative cell viability of human cancer cells after 48-hour incubation with kava extracts at 75 µg/mL (red, circles) and 150 µg/mL (blue squares) is plotted to sample FLK B concentrations (log_2_ normalized) with R^2^ values shown at the top for each extract concentration.
(EPS)

Table S1 **Commercial Kava Sources.**
(DOCX)

Table S2 **Average concentration (ppm) of compounds from dry powder commercial kava sources.**
(DOCX)

Table S3 **Average concentration (ppm) of compounds from liquid commercial kava sources.**
(DOCX)
