# Supplementary material for: Measuring the Chemical and Cytotoxic Variability of Commercially Available Kava (Piper methysticum G. Forster)
Source: PLoS One. 2014 Nov 3;9(11):e111572. doi: 10.1371/journal.pone.0111572 (PMC4218769; doi:10.1371/journal.pone.0111572)
Supplement: Table S1 — Commercial Kava Sources. (DOCX) [file pone.0111572.s005.docx]

| **Table S1. Commercial Kava Sources** | | | | |
| --- | --- | --- | --- | --- |
| **Name** |  | **Type^A^** |  | **Source, harvest date, origin, and notable properties** |
| Black Sand Kava Nakamal Grade |  | P |  | [Nakamal@Home](mailto:Nakamal@Home), June 2011 Republic of Vanuatu; fine grind |
| Kava powder P.E. 30% |  | P |  | December 2010; very fine grind |
| Freeze-dried Borogoru kava |  | P |  | Ed Johnston, Hawaii, USA; freeze-dried**^B^** |
| Tanna Kava |  | P |  | Tanna Kava Kava from the Jungle; coarse grind |
| Fire Island Kava Instant Kava, 100% dried kava juice |  | P |  | [Nakamal@Home](mailto:Nakamal@Home), Republic of Vanuatu; freeze-dried**^B^** |
| Kava Kava extract with glycerin and grain alcohol |  | L |  | Now Foods, Republic of Vanuatu/Fiji; glycerin extract |
| Solomon Kava Nambawan (#1) grade |  | P |  | [Nakamal@Home](mailto:Nakamal@Home), June 2011, Solomon Islands; very fine grind |
| Stone Kava Stone grade |  | P |  | [Nakamal@Home](mailto:Nakamal@Home), June 2011 Republic of Vanuatu; fine grind |
| Big Island Grown Kava |  | P |  | Paradise Kava, June 2011, Hawaii, USA; fine grind |
| Kava Kava Root with grain alcohol, glycerin, and water |  | L |  | Gaia Herbs, Republic of Vanuatu; medium to high alcohol extract |
| Kava Kava critical CO_2_ extract |  | L |  | Mr. Jay Stopper |
| Pharma Kava® Liquid Extract with grain alcohol |  | L |  | Herb Pharm, Republic of Vanuatu; “organic” grain alcohol extract |
| Fiji Kava Bula Grade |  | P |  | [Nakamal@Home](mailto:Nakamal@Home) June 2011, Fiji; fine grind |
| Whole kava root |  | P |  | Medium fine grind |
| Kava Kava Root with grain alcohol and water |  | L |  | Gaia Herbs, Republic of Vanuatu; high alcohol extract |
| Kava extract with 95% ethanol |  | L |  | Kennin Garrett, August 2001 |
| Kava professional Gaia Herb |  | L |  | Medium to high alcohol |
| Pentecost Pride kava |  | P |  | Vanuatu Kava Store, Republic of Vanuatu; medium fine grind |
| Wow! Kava Connoisseur Grade |  | P |  | [Nakamal@Home](mailto:Nakamal@Home), June 2011 Republic of Vanuatu; fine grind |
| Fire Island Kava Instant Kava, 100% dried/ground kava roots |  | P |  | [Nakamal@Home](mailto:Nakamal@Home), Republic of Vanuatu; freeze-dried**^B^** |
| Dry kava root |  | P |  | Kennin Garrett; coarse grind |
| Kava Kava Root with glycerin, water, and <8% grain alcohol |  | L |  | Gaia Herbs; low alcohol extract |
| Kava water, homemade, 100% aqueous |  | L |  | Ed Johnston, Hawaii, USA; alcohol-free |
| Kava Tincture with 97% ethanol |  | L |  | Hilo Natural Health Clinic, Hawaii, USA |
| All products were obtained in fall of 2011, the harvest date and country of origin is indicated for products where this information was available. (A) Type describes the starting state of the source material P- dry powder and L-liquid. (B) Extracted kava was freeze-dried and pulverized to form a water soluble instant drink mix. | | | | |
