# Supplementary material for: Measuring the Chemical and Cytotoxic Variability of Commercially Available Kava (Piper methysticum G. Forster)
Source: PLoS One. 2014 Nov 3;9(11):e111572. doi: 10.1371/journal.pone.0111572 (PMC4218769; doi:10.1371/journal.pone.0111572)
Supplement: Table S2 — Average concentration (ppm) of compounds from dry powder commercial kava sources. (DOCX) [file pone.0111572.s006.docx]

| **Table S2.** **Average concentration (ppm) of compounds from dry powder commercial kava sources** | | | | | | | |
| --- | --- | --- | --- | --- | --- | --- | --- |
| Commercial Source Codes | Extraction method | K | DHK | M | DHM | FLK A | FLK B |
| C | I | 9 ±2 | 22 ±1 | 2.0 ±0.2 | 6.7 ±0.6 | ND | ND |
|  | II | 30 ±1 | 36 ±1 | 17.3 ±1.8 | 26.9 ±0.7 | 2.7 ±0.2 | 2.1 ±0.2 |
| G | I | 13.3 ±0.6 | 23.4 ±0.4 | 2.9 ±0.2 | 8.1 ±0.3 | ND | ND |
|  | II | 45 ±3 | 35 ±1 | 21 ±3 | 25 ±1 | 5.3 ±1.1 | 6.2 ±1.4 |
| H | I | 13 ±1 | 22.9 ±0.5 | 2.7 ±0.2 | 8.1 ±0.4 | 0.04 ±0.01 | 0.02±0.01 |
|  | II | 42 ±1 | 34.9 ±0.5 | 13.2 ±1.6 | 25 ±1 | 4.9 ±0.8 | 5.5 ±0.9 |
| P | I | 47.2 ±2.4 | 36.8 ±0.3 | 19.3 ±2.1 | 17.3 ±0.1 | ND | ND |
|  | II | 25.6 ±0.3 | 34.0 ±0.8 | 8.1 ±0.2 | 19.1 ±0.9 | 3.1 ±0.6 | 3.1 ±0.3 |
| Q | I | 11 ±1 | 24.6 ±1.0 | 1.9 ±0.2 | 8.2±0.8 | 0.02 ±0.01 | 0.01±0.008 |
|  | II | 51 ±2 | 38.1 ±0.5 | 30.4 ±3.6 | 30 ±1.0 | 8.4 ±0.8 | 10±1 |
| R | I | 11.2 ±0.7 | 22.4 ±0.8 | 2.4 ±0.2 | 7.8 ±0.5 | 0.04 ±0.01 | 0.04±0.004 |
|  | II | 51 ±1 | 37 ±1 | 32.2 ±1.5 | 28.5 ±0.4 | 6 ±1 | 7.5 ±0.7 |
| S | I | 15 ±2 | 28.3 ±1.9 | 3.7 ±0.7 | 12 ±2 | 0.02±0.01 | 0.02±0.008 |
|  | II | 50 ±1 | 37.3 ±0.9 | 29 ±5 | 30.1 ±0.7 | 7 ±1 | 7.4 ±1.2 |
| T | I | 10.9 ±0.2 | 22.8 ±0.3 | 3.3 ±0.1 | - 1. ±0.2 | ND | 0.005±0.003 |
|  | II | 48.3 ±3.6 | 38 ±1 | 24 ±2 | 22.1 ±0.6 | 4.4 ±0.5 | 5.7 ±0.7 |
| U | I | 11.3 ±0.3 | 21.7 ±0.4 | 2.6 ±0.1 | 6.1 ±0.2 | ND | ND |
|  | II | 41 ±6 | 34.5 ±0.5 | 21.2 ±4.5 | 22 ±1 | 2.4 ±0.9 | 3.3 ±1.1 |
| V | I | 18.4 ±0.2 | 30.6 ±0.3 | 5.2 ±0.1 | 12.8 ±0.2 | ND | ND |
|  | II | 65.3±4.3 | 38.2 ±0.7 | 41.4 ±3.3 | 27 ±1 | 5.9 ±0.3 | 5.7 ±0.2 |
| W | I | 12 ±1 | 17.4 ±0.7 | 4.1 ±0.6 | 6.4 ±0.6 | 0.05 ±0.006 | 0.1 ±0.01 |
|  | II | 45±2 | 36.7 ±0.5 | 25 ±4 | 24.7 ±0.3 | 2.7 ±0.4 | 3.6 ±0.5 |
| Z | I | 26.4 ±0.4 | 35.7 ±0.4 | 6.4 ±0.2 | 20.8 ±0.3 | ND | 0.01±0.001 |
|  | II | 45 ±1 | 38.3 ±0.4 | 6.4 ±0.2 | 33 ±1 | 13.1 ±0.6 | 14.7±0.7 |
| AA | I | 11 ±2 | 20 ±2 | 2.4 ±0.4 | 6.7 ±0.9 | ND | ND |
|  | II | 26.5 ±0.2 | 25.5 ±0.4 | 13.4 ±1.0 | 19.7 ±0.4 | 1.3 ±0.2 | 0.6 ±0.07 |
| CC | I | 23.2±6.9 | 27 ±3 | 7.5 ±2.8 | 13.8 ±4.0 | 1.6 ±0.6 | 1.6 ±0.8 |
|  | II | 41 ±1 | 33.9 ±0.5 | 14.3 ±2.1 | 23.0 ±0.7 | 2.2 ±0.3 | 2.5 ±0.3 |
| Values represent the mean of four extraction replicates with standard error reported. ND indicates that the concentration was below the level of detection. Extraction method I used water and method II used 95% ethanol. Compounds are abbreviated as follows: K - kawain, DHK - dihydrokawain, M - methysticin, DHM - dihydromethysticin, FLK A, - flavokawain A, FLK B - flavokawain B. | | | | | | | |
