# Supplementary material for: Measuring the Chemical and Cytotoxic Variability of Commercially Available Kava (Piper methysticum G. Forster)
Source: PLoS One. 2014 Nov 3;9(11):e111572. doi: 10.1371/journal.pone.0111572 (PMC4218769; doi:10.1371/journal.pone.0111572)
Supplement: Table S3 — Average concentration (ppm) of compounds from liquid commercial kava sources. (DOCX) [file pone.0111572.s007.docx]

| **Table S3.** **Average concentration (ppm) of compounds from liquid commercial kava sources** | | | | | | | |
| --- | --- | --- | --- | --- | --- | --- | --- |
| Commercial Source Codes | Extraction method | K | DHK | M | DHM | FLK A | FLK B |
| K | III | 4.2 ±1.2 | 6.6 ±1.4 | 1.4 ±0.5 | 2.1 ±0.6 | ND | 0.01±0.005 |
|  | IV | 31 ±1.0 | 29.3 ±0.1 | 11.3 ±0.0 | 16.9 ±0.2 | 0.5 ±0.01 | 0.7 ±0.007 |
| L | III | 2.1 ±0.8 | 3.2 ±1.1 | 0.3 ±0.2 | 0.8 ±0.4 | ND | ND |
|  | IV | 37.5 ±0.1 | 32.3 ±0.2 | 9.6 ±0.4 | 18.6 ±0.3 | 0.09±0.01 | 0.1 ±0.01 |
| M | III | 0.08±0.1 | 0.2 ±0.0 | 0.03±0.0 | 0.02 ±0.00 | ND | ND |
|  | IV | 19±3.4* | 21±3* | 6±1.3* | 9.4±1.5* | 0.04±0.01* | 0.07±0.06* |
| N | III | 2.3 ±0.4 | 4.3 ±0.5 | 0.5 ±0.1 | 1.1 ±0.2 | ND | ND |
|  | IV | 16±6 | 18.2 ±9.7 | 7 ±3 | 12 ±2 | 8.5 ±0.2 | 5.0 ±0.6 |
| O | III | 9 ±3 | 12.5 ±2.8 | 5 ±2 | 6 ±2 | 0.06±0.03 | 0.06±0.02 |
|  | IV | 26±4.7* | 31±4.3* | 10±2.1* | 18±2.6* | 0.03±0.004* | 0.06±0.01* |
| X | III | 32±6 | 34 ±1 | 12 ±7 | 19 ±2 | 0.9 ±0.09 | 0.3 ±0.1 |
|  | IV | 40±7.2* | 44±6.1* | 14±3* | 26±4.1* | 0.3±0.04* | 1.2 ±0.3* |
| Y | III | 27 ±1 | 28 ±1 | 9 ±1 | 14.2 ±0.6 | 0.07 ±0.1 | 0.04±0.02 |
|  | IV | 20±13 | 24 ±4 | 8 ±6 | 12 ±6 | 1.1 ±0.6 | 0.6 ±0.3 |
| BB | III | 33±9 | 36.2 ±0.4 | 13 ±6 | 28.3 ±0.1 | 2.1 ±0.05 | 0.9 ±0.009 |
|  | IV | 49.4 ±0.7 | 33 ±4 | 17±1 | 24 ±6 | 6.9 ±0.5 | 7.2 ±0.5 |
| DD | III | 5 ±2 | 6.4 ±2.0 | 0.5 ±0.2 | 2 ±1 | ND | ND |
|  | IV | 24±4.3* | 27±3.8* | 3.8±0.8* | 13±2.1* | 0.02±0.003* | 0.07±0.2* |
| EE | III | 17.0 ±0.1 | 23.9 ±0.2 | 3.5 ±0.1 | 10.0 ±0.1 | ND | ND |
|  | IV | 22.4 ±0.2 | 27.0 ±0.1 | 5.9 ±0.1 | 14.4 ±0.1 | ND | ND |
| Values represent the mean of four extraction replicates with standard error reported. *Single replicate analysis with error estimates based on calculated average relative error. ND indicates that the concentration was below the level of detection. Extraction method III used water and method IV used 95% ethanol. Compounds are abbreviated as follows: K - kawain, DHK - dihydrokawain, M - methysticin, DHM - dihydromethysticin, FLK A, - flavokawain A, FLK B – flavokawain B. | | | | | | | |
